# Molecular Detection and Phylogenetic Analysis of the alkB Gene in *Klebsiella oxytoca* Strains Isolated from the Gut of *Tenebrio molitor*

**DOI:** 10.1155/2024/3350591

**Published:** 2024-05-09

**Authors:** Tsitsi Lynn Mupamhadzi, Oleen Machona, Farisai Chidzwondo, Rumbidzai Mangoyi

**Affiliations:** Department of Biotechnology and Biochemistry, University of Zimbabwe, Harare, Zimbabwe

## Abstract

The challenge in polystyrene disposal has caused researchers to look for urgent innovative and ecofriendly solutions for plastic degradation. Some insects have been reported to use polystyrene as their sole carbon source, and this has been linked to the presence of microbes in their guts that aid in plastic digestion. Thus, this study focuses on the molecular detection and phylogenetic analysis of the alkane-1-monooxygenase (alkB) gene in *Klebsiella oxytoca* strains isolated from the gut of *Tenebrio molitor*. The alkB gene encodes for alkane-1-monooxygenase, an enzyme involved in the oxidation of inactivated alkanes. This gene can be used as a marker to assess bacteria's ability to biodegrade polystyrene. Three bacterial strains were isolated from the guts of *T. molitor* mealworms and were confirmed using polymerase chain reaction (PCR) of the 16S ribosomal RNA gene. The primers used in the amplification of the 16S ribosomal RNA region were designed using NCBI, a bioinformatics tool. To detect the presence of the alkB gene in the isolated bacterial strains, a set of primers used in the amplification of this gene was manually designed from the conserved regions of the alkB nucleotide sequences of eleven bacterial species from GenBank. TCOFFE online tool was used to align the alkB sequences of the bacteria, while Jalview and ConSurf were used to view the alignment. The amplified alkB gene was then sequenced using the Sanger sequencing technique, blasted on NCBI to look for similar sequences, and a phylogenetic tree was constructed. Based on the 16S ribosomal RNA gene sequences, the isolated bacterial strains were confirmed to be *Klebsiella oxytoca* NBRC 102593, *Klebsiella oxytoca* JCM 1665, and *Klebsiella oxytoca* ATCC 13182. The alkB gene sequence identical to fourteen alkB gene sequences derived from *Actinobacteria* whole genome was detected in *Klebsiella oxytoca* for the first time to the best of our knowledge. The novel nucleotide sequence was published in the NCBI database under accession number OP959069. This gene sequence was found to be for the enzyme alkane-1-monooxygenase and may be one of the enzymes responsible for polystyrene degradation by the putative *Klebsiella oxytoca* ATCC 13182 in *T. molitor*.

## 1. Introduction

*Tenebrio molitor* is the larval form of the yellow mealworm beetle that is known as the darkling beetle [1]. The mealworms are well-known scavengers and decomposers of the earth's surface. They consume decaying leaves, sticks, grasses, plants, insects, and animals. This behaviour has been found to be beneficial to ecology because they devour organic matter that other organisms do not [2]. These include plastic materials such as polycarbonate (PC), polystyrene (PS), and expanded polystyrene (EPS) among others. Because of this characteristic, researchers have identified mealworms as a possible polystyrene-degrading insect [3].

Many studies have demonstrated that the tendency to eat plastic in these insects might be associated with the digestive activity of intestinal microorganisms, which would help them with the digestion process [4]. Research is therefore increasingly focusing on bacterial species isolated from plastic-degrading larvae and their potential to digest plastic on their own. Three bacterial species from the intestines of *T. molitor* were reported to be good plastic biodegraders [5]. These bacterial species were *Serratia marcescens*, *Klebsiella oxytoca*, and *Pseudomonas aeruginosa*. In a more recent study, three bacterial isolates identified as *Klebsiella oxytoca* NBRC 102593 strain*, Klebsiella oxytoca* JCM 1665 strain, *and Klebsiella oxytoca* ATCC 13182 strain were isolated from *T. molitor* mealworms which were fed solely on polystyrene for 7 days [6]. From this study, it was then concluded that the *Klebsiella oxytoca* species are among the species responsible for the biodegradability of waste-expanded polystyrene by *Tenebrio molitor* mealworms.

The *Klebsiella oxytoca* is an enteric bacterium which is a Gram-negative member of the *Klebsiella* family. Recent studies have investigated the use of *Klebsiella oxytoca* strains as enzyme sources for plastic biodegradation, alkane hydroxylases (AHs) being the key enzyme [7]. Monooxygenases are the most important enzymes in the alkane hydroxylase system. Thus far, the integral-membrane alkane monooxygenase (alkB)-related AHs are the most common AHs found in both Gram-negative and Gram-positive bacteria [8].

The aim of this study was to investigate the possible presence of the alkane-1-monooxygenase (alkB) gene responsible for polystyrene degradation, in *Klebsiella oxytoca* strains isolated from the gut of *T. molitor*.

## 2. Materials and Methods

### 2.1. Isolation of *Klebsiella oxytoca* Strains from *T. molitor's* Gut

A total number of 50 *Tenebrio molitor* mealworms were fed with polystyrene as the sole carbon source for seven days and then sterilized by immersing them in 75% alcohol for one minute. The mealworms were immersed in saline water (0.85%) for one minute. Saline water was used to remove the alcohol and kill the mealworms by osmosis as it contains salt. Surgical blades and forceps were used to remove the entire gut of the worms. The guts were placed in a sterile Petri dish, and the midguts were drawn out and placed in a centrifuge tube containing 5 ml of saline solution. A Dounce homogenizer was then used to homogenize the sample for 10 minutes. The gut cell suspension was collected and inoculated into a flask containing 100 ml of polystyrene emulsion and liquid carbon-free basal medium (LCFBM). The flask that served as the control contained polystyrene emulsion and LCFBM only. The flasks were then incubated on a rotary shaker at 120 rpm at 30 degrees for 28 days. After 28 days, the solution was spread on a plate with modified polystyrene agar. The plate was incubated for 24 hours at 30 degrees to increase the number of bacteria present in the culture. Colonies from the plate were collected based on morphology and subcultured on fresh polystyrene agar. The quadrant streak method was used to obtain pure colonies.

## 3. Molecular Identification of the Bacteria

### 3.1. Designing of Primers

Confirmation of isolation of *Klebsiella oxytoca* strains, *Klebsiella oxytoca* ATCC 13182*, Klebsiella oxytoca* JCM 1665, *and Klebsiella oxytoca* NBRC 102593 was done using polymerase chain reaction (PCR). Primers that amplify the 16S ribosomal RNA gene in these bacterial strains were designed using the sequences of the 16S ribosomal RNA gene for the three bacterial strains. The sequences used to design the primers were previously reported [6]. To design the primers, the NCBI primer blast tool (https://www.ncbi.nlm.nih.gov/tools/primer-blast/index.cgi) was used. Each of these sequences was uploaded to the NCBI Primer blast tool. A PCR product size of 70 up to a thousand base pairs was specified. The melting temperature of each primer was set to a minimum of 50°C and a maximum of 63°C with an optimum of 60°C, and the maximum melting temperature difference between the 2 primers was set to 3°C. The GC content for the primers was set between 45% and 60%. More than ten sets of primers were returned after blasting. A pair for each strain that had the right characteristics of appropriate primers was selected. An offline tool, SnapGene viewer (https://www.snapgene.com/snapgene-viewer), was used for in silico PCR to note the expected amplicon sizes. Lyophilized primers were ordered from Inqaba Biotec and were diluted to a stock concentration of 100 mM using the protocol provided for each primer. The stock solutions were further diluted to make a working solution of 10 mM using nuclease-free sterile water.  Table 1 shows the sequences used to design the primers.

### 3.2. Amplification of the 16S Ribosomal RNA Gene by PCR

Conventional PCR was carried out using the designed primers to amplify the 16S ribosomal RNA gene in each of the 3 isolates obtained. To prepare the DNA template for the PCR, single colonies were either picked from the culture plates and crushed in 10 *μ*L of nuclease-free water, and the crushed samples were incubated at 95°C for 10 minutes and centrifuged at 13000 rpm for two minutes or cultured overnight in LB broth, using 1 *μ*L of the overnight culture as a template. Each of the isolate suspensions was used as a DNA template for amplification using all the designed primer sets in 3 different reactions to determine which of the strains was *Klebsiella oxytoca* ATCC 13182, *Klebsiella oxytoca* JCM 1665, and *Klebsiella oxytoca* NBRC 102593. PCR reactions (10 *μ*L) were prepared by mixing 5 *μ*L of One Taq® Quick-Load® 2X Master Mix with Standard Buffer supplied by New England Biolabs (https://www.neb.com/M0486) with 1 *μ*L each of forward and reverse primers, 2 *μ*L nuclease free water, and 1 *μ*L of the template DNA. Cycling conditions for conventional PCR for all the primers were as follows: one cycle of the initial denaturation at 95°C for 10 minutes followed by 30 cycles of 95°C for 30 seconds, primer annealing at 55°C for 30 seconds, extension at 72°C for 30 seconds, and a final extension at 72°C for 10 minutes, and samples were cooled to 4°C.

### 3.3. Gel Electrophoresis to View PCR Results

The SnapGene tool was used in *in silico* gel electrophoresis. Different conditions were specified for the *in silico* gel to determine the optimum gel electrophoresis conditions. Guided by the *in silico* gel, a 1% agarose gel was prepared by dissolving 1 gram of agarose in 100 ml of 1 X TAE buffer and microwaving the mixture for 2 minutes with intermittent mixing. The agarose solution was allowed to cool to about 50°C before 3 *μ*L of ethidium bromide was added. The solution was then poured into a gel tank and left to set for 15 minutes. Amplicons and the molecular weight marker were loaded into each well on the agarose gel. 100 bp and 1 kb genomic ladders were used in gel electrophoresis for PCR reaction with JCM primers and NBRC primers, respectively. Gel electrophoresis was done for 20 minutes at a voltage of 100 A. A UV transilluminator was used for the visualization of the band pattern on the agarose gel.

### 3.4. Molecular Detection of the Alkane 1-Monooxygenase (alkB) Gene in Each Strain

Eleven alkB gene sequences from eleven different bacterial strains from GenBank were selected for designing the primers. The accession numbers of the selected sequences are given in Table 2. These sequences were then aligned using TCOFFE (https://www.ebi.ac.uk/Tools/msa/tcoffee/). To view the alignment, Jalview, an offline tool, and ConSurf (The ConSurf Server (tau.ac.il)), an online tool, were used. These tools made it easy to determine the conserved and variable regions of the alignment clearer. Primers were designed from the conserved regions to have a GC content between 45% and 60%, *T*_*m*_ between 60°C and 62°C, and length between 18 and 24 bp. The forward primer was designed from bases 210 to 230 of the alignment. The reverse primer was designed from bases 540 to 520 of the alignment. SnapGene was used to note the attachment of the primers to the template. The designed primers were named Deg-r for the reverse and Deg-f for the forward. Lyophilized primers were ordered from Inqaba Biotec, dissolved and diluted to a stock concentration of 100 mM using the protocol provided for each primer. The stock solutions were further diluted to make a working solution of 10 mM using nuclease free sterile water.

### 3.5. Selection of Other alkB Targeting Primers

To add to the set of primers designed, two more sets were selected from the literature [9]. These primers were successful in amplifying the gene in 49% of the bacteria in the study. The selected primers were Alkb-f and Alkb-r and Alk-BFB and Alk-BRB.

### 3.6. Molecular Detection of the alkB Gene

To detect the presence of the alkB gene in the bacteria of *Klebsiella* strains, touchdown colony PCR was performed on all the samples against each of the 3 primer sets, Alkb-f and Alkb-r, Alk-BFB and Alk-BRB, and the designed Deg-f and Deg-r. Cycling conditions for Deg-f and Deg-r were as follows: one cycle of 95°C for 10 minutes, 20 cycles of 95°C for 2 minutes, 55°C for 1 minute (annealing), and 72°C for 30 seconds, reducing the annealing temperature by 0.5°C each cycle, and 20 cycles of 95°C for 2 minutes, 45°C for 1 minute (annealing) and 72°C for 30 seconds, and final extension at 72°C for 10 minutes and final hold at 4°C∞. For Alkb-f and Alkb-r primer sets, the cycling conditions were as follows: one cycle of 95°C for 10 minutes, 20 cycles of 95°C for 2 minutes (denaturation), 65°C for 1 minute (annealing), and 72°C for 30 seconds (extension), reducing the annealing temperature by 0.5°C each cycle, and 20 cycles of 95°C for 2 minutes (denaturation), 55°C for 1 minute (annealing) and 72°C for 30 seconds (extension), and final extension at 72°C for 10 minutes, and samples were cooled to 4°C. For Alk-BFB and Alk-BRB primer sets, the cycling conditions were as follows: one cycle of 94°C for 3 minutes, 16 cycles of 94°C for 30 seconds, 61°C for 30 seconds (annealing), and 72°C for 30 seconds, reducing the annealing temperature by 0.5°C each cycle, and 24 cycles of 94°C for 30 seconds, 53°C for 30 seconds (annealing) and 72°C for 30 seconds, and a final extension at 72°C for 10 minutes and final hold at 4°C∞.

PCR products were separated by 1% agarose gel electrophoresis and visualized. A 100 bp genomic ladder was used in gel electrophoresis for PCR reaction with Alk-BFB and Alk-BRB, and Deg-f and Deg-r. 1 kb genomic ladder was used in gel electrophoresis for a PCR reaction with Alkb-f and Alkb-r. Gel electrophoresis was done for 20 minutes at a voltage of 100 A. A UV transilluminator was used for visualization of the agarose gel.

### 3.7. Sequencing and Analysis of the PCR Products

The products of the genes that were successfully amplified were sent to a local company called Biotech Research Institute for sequencing using the Sanger sequencer. A DNA sequence reader CHROMAS (https://chromas.software.informer.com/2.5/) was downloaded and used to view and analyse the sequence. The analysis included identification and trimming of low-quality bases and manual base calling of degenerate bases to obtain a good sequence.

The cleaned sequence was saved in FASTA format in a word document. The FASTA sequence was blasted on NCBI against nucleotide sequences in GenBank.

### 3.8. Phylogenetic Analysis

AlkB gene sequences from 14 different bacteria were chosen from GenBank. These sequences were used for phylogenetic analysis in MegaX. The evolutionary relationship in MegaX was inferred by using the maximum likelihood method and Tamura–Nei model.

## 4. Results

### 4.1. Isolation of the Polystyrene-Degrading *Klebsiella oxytoca* Strains from *T. molitor's* Gut

**Figure 1:**
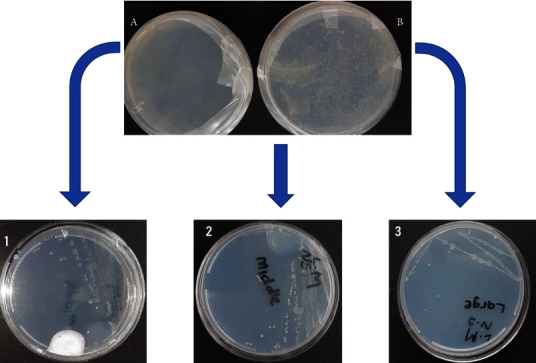
Overnight cultures of the bacteria isolated from the midgut of the *T. molitor* worms. A is the control which is the culture medium only, and B is the isolated bacteria on the culture media, and 1, 2, and 3 are the pure colonies obtained after the quadrant streak method.

The colonies obtained after culturing *Tenebrio's* gut cell suspension in modified polystyrene agar were collected based on the morphology of the *Klebsiella* oxytoca strains and subcultured on fresh polystyrene agar using the quadrant streak method to obtain pure colonies. Three different isolates were identified, and these were reported as samples 1, 2, and 3 in Figure 1. Sample 1 colonies were round, cream, and shiny. These colonies were the smallest as compared to the other colonies in samples 2 and 3. Sample 2 colonies were white and elongated. Sample 3 colonies were whitish and were the biggest among all the 3 colonies.

## 5. Molecular Identification of the Bacteria

### 5.1. Designing of Primers

To confirm that the isolated bacteria were *Klebsiella oxytoca* strains, primers that amplify the 16S ribosomal RNA gene in the three *Klebsiella* strains were designed using the sequences of the 16S ribosomal RNA gene. Primers that amplify the 16S ribosomal RNA region of *Klebsiella oxytoca* NBRC 102593 were designed to give a product of 247 base pairs. The GC content of the forward and the reverse primers for this primer set was 55% and 60%, respectively. The melting temperatures were 59.96°C and 60.53°C, respectively. The self-complementarity score of the forward and reverse primers was 4 and 5, and the 3′ complementary score was 0 and 3. Primers that amplify the 16S ribosomal RNA region of *Klebsiella oxytoca* ATCC 13182 strain were designed to give a product of 329 base pairs. The GC content of the forward and the reverse primers for this primer set was 52.6% and 55%, respectively. The melting temperatures were 57.6°C and 59.9°C, respectively. The self-complementarity score of the forward and reverse primers was 4 and 3, and the 3′ complementary score was 2 and 3. Primers that amplify the 16S ribosomal RNA region of *Klebsiella oxytoca* JCM 1665 were designed to give a product of 262 base pairs. The GC content of the forward and the reverse primers for this primer set was 60% and 50%, respectively. The melting temperatures were 60.6°C and 61.46°C, respectively. The self-complementarity score of the forward and reverse primers was 4 and 6, and the 3′ complementary score was 3 and 1.  Table 3 shows the forward and the reverse primer sequences designed to amplify the 16S ribosomal RNA of the *Klebsiella oxytoca* NBRC 102593 strain*, Klebsiella oxytoca* JCM 1665 strain, and *Klebsiella oxytoca* ATCC 13182 strain.

### 5.2. Amplification of the 16S Ribosomal RNA Gene by PCR

Conventional PCR was carried out using the designed primers to amplify the 16S ribosomal RNA gene in each of the 3 isolates obtained. Each of the isolates was amplified using all the 3 designed primer sets to determine which of the strains was *Klebsiella oxytoca* ATCC 13182*, Klebsiella oxytoca* JCM 1665, and *Klebsiella oxytoca* NBRC 102593.

**Figure 2:**
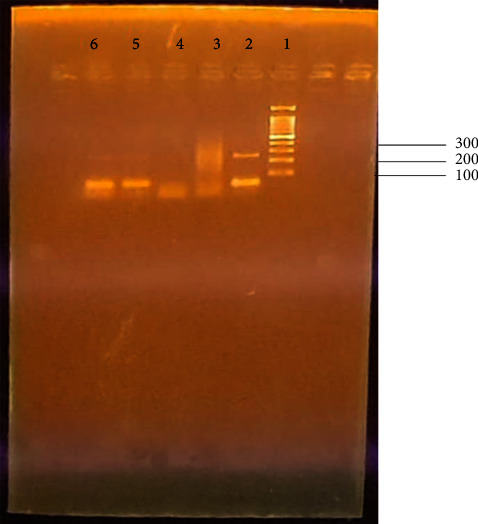
Amplification of the three *Klebsiella oxytoca* strains with JCM-specific primers on 1.5% agarose gel. Lane 1:100 bp ladder, lane 2: sample 1 which was identified as *Klebsiella oxytoca* JCM 1665, lane 3: positive control which is the master mix with 16S ribosomal RNA primers plus the DNA template, lane 4: negative control which is the PCR master mix without the DNA template, and lanes 5 and 6 had samples 2 and 3, respectively.

Figure 2 shows amplification of the three *Klebsiella oxytoca* strains with JCM-specific primers on 1.5% agarose gel. The JCM primers which were used in this reaction to amplify *Klebsiella oxytoca* JCM 1665 strain had an expected product size of 262 base pairs. The gel shows a band at an approximate size of 260 base pairs in well number 2 which was loaded with sample 1. Thus, sample 1 was identified as *Klebsiella oxytoca* JCM 1665. Only primer dimers above 100 bp mark were observed in wells 5 and 6 (loaded with samples 2 and 3, respectively).

**Figure 3:**
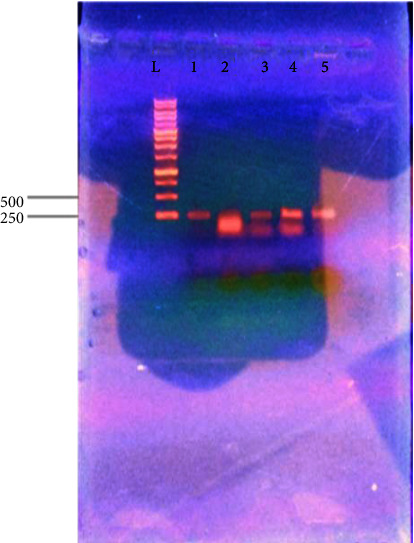
Amplification of the *Klebsiella oxytoca* NBRC 102593 strain with NBRC-specific primers on a 1.5% agarose gel. Lane L-1 kb ladder, lane 2: negative control, lanes 1 and 3: sample 2 in LB broth overnight culture, and lanes 4 and 5: sample 2 as single colonies from culture plates. The results as shown in all the 4 lanes show a band of approximately 247 base pairs, which was the expected product size with NBRC primers.

Figure 3 shows amplification of *Klebsiella oxytoca* NBRC 102593 strain with NBRC-specific primers on a 1.5% agarose gel when 1 *μ*L of LB broth overnight culture was used as the DNA template and when single colonies were picked from overnight culture plates and used as a DNA template for sample 2. The results show that sample 2 was identified as *Klebsiella oxytoca NBRC* 102593 strain since all 4 lanes show a band of approximately 247 base pairs, which was the expected product size with NBRC primers. The results also show that the two forms of the template DNA work in a PCR reaction in the same way.

Amplification of the three *Klebsiella oxytoca* strains with ATCC-specific primers was also carried out and the expected product size of 337 base pairs was observed in the well loaded with sample 3.

Thus, from the results obtained from the amplification of the 16S ribosomal RNA gene by PCR, the isolated samples were identified as *Klebsiella oxytoca* JCM 1665 strain—sample 1, *Klebsiella oxytoca* NBRC 102593 strain—sample 2, and *Klebsiella oxytoca* ATCC 13182 strain—sample 3.

## 6. Molecular Detection of the Alkane-1-Monooxygenase (alkB) Gene in *Klebsiella oxytoca* Strain

### 6.1. Designing of Oligonucleotide Primers That Amplify alkB Gene

To detect the presence of the alkB gene in each of the three isolates, primers that amplify the alkB gene were designed manually.  Table 3 shows the forward and the reverse (Deg-f and Deg-r degenerate) primer sequences designed to amplify the alkB gene in the three K*lebsiella oxytoca* strains. The primers that amplify the alkB gene in *Klebsiella oxytoca* strains were designed to give a product size of 338 base pairs. The forward primer had a *T*_*m*_ of 42.81°C, a GC content of 50%, a self-complementary score of 4 and 3′ complementary score of 0. The reverse primer had a *T*_*m*_ of 47.69°C, a GC content of 58.82%, a self-complementary score of 6 and 3′ complementary score of 0.

### 6.2. Oligonucleotide Primers Chosen from Literature That Amplify the alkB Gene

To increase the chances of amplifying the alkB gene in the three isolates, two more primer sets were selected from the literature to add to the one which was designed to make them 3 primer sets. The sequences of the 2 sets of primers selected from the literature are shown in Table 3. The selected sets of primers were AlkB-f and AlkB-r degenerate and Alk-BFB and Alk-BRB.

### 6.3. Amplification of the alkB Gene by PCR in *Klebsiella oxytoca* Strains

**Figure 4:**
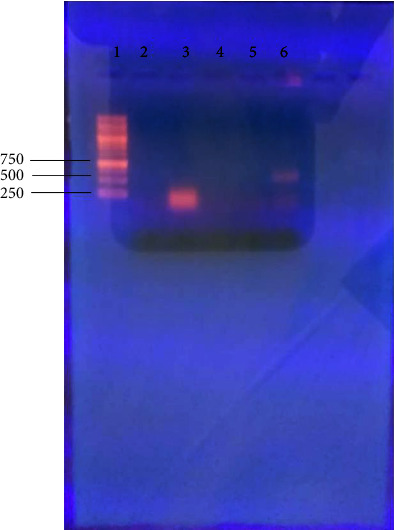
PCR amplification of the three *Klebsiella oxytoca* strains with AlkB-f and AlkB-r degenerate primers on 1.5% agarose gel. Lane 1: 1 kb ladder, lane 2: negative control, lane 3: positive control, lane 4: sample 1, lane 5: sample 2, and lane 6: sample 3.

Figure 4 shows the results of the PCR amplification of the three *Klebsiella oxytoca* strains with AlkB-f and AlkB-r primers on 1.5% agarose gel. Amplification was observed in lane 6 as shown by a band on the gel at approximately 550 base pairs, an expected product size of the alkB gene with AlkB-f and AlkB-r primers. Lane 6 contained sample 3. However, no bands were observed in other lanes (2–5) confirming no amplification of the gene in samples 1 and 2. Sample 3 had already been identified as *Klebsiella oxytoca* ATCC 13182 by amplification of 16S using PCR.

**Figure 5:**
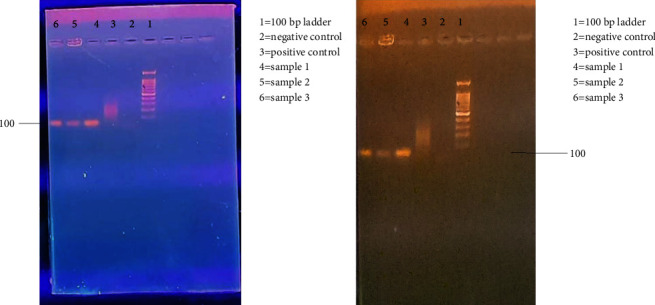
Amplification of the three *Klebsiella oxytoca* strains with (a) Deg-f and Deg-r degenerate primers and (b) Alk-BFB and Alk-BRB primers on 1.5% agarose gel. Lane 1: 100 bp ladder, lane 2: negative control, and lane 3: positive control. Lanes 4–6: samples 1, 2, and 3, respectively.

Figure 5 shows the result of the PCR amplification of the three *Klebsiella oxytoca* strains with Alk-BFB and Alk-BRB degenerate primers and Deg-f and Deg-r primers on 1.5% agarose gel. These primers could not amplify the gene since no bands were observed in the lanes. The gels show primer dimers only which are just above the 100 base pair mark and no band at the expected level on the gel.

Thus, results from amplification of the alkB gene by PCR showed that only sample 3 which had been identified as *Klebsiella oxytoca* ATCC 13182 strain contained this alkB gene.

## 7. Sequencing of the Gene and Sequence Analysis

### 7.1. Gene Sequencing

The PCR product from the *Klebsiella oxytoca* ATCC 13182 strain was sequenced to confirm if the amplified gene was the expected alkB gene.

### 7.2. Sequence Analysis

The query nucleotide sequence, which is the alkB sequence in *Klebsiella oxytoca*, was then blasted against nucleotide sequences in GenBank in search for homology, and the results obtained from NCBI are shown in Table 4. The first column of the table shows the sequence accession number for the whole genome of the bacteria in the alignment, the third column shows the percentage identity of the query sequence, and the last column shows a link to the exact region within the genome of the bacteria that was involved in the alignment which is the alkB gene in all ten genomes. The alkB sequence in *Klebsiella oxytoca* ATCC strain was found to have more than 95% similarity with the alkB gene sequences in these 10 bacteria species.

**Figure 6:**
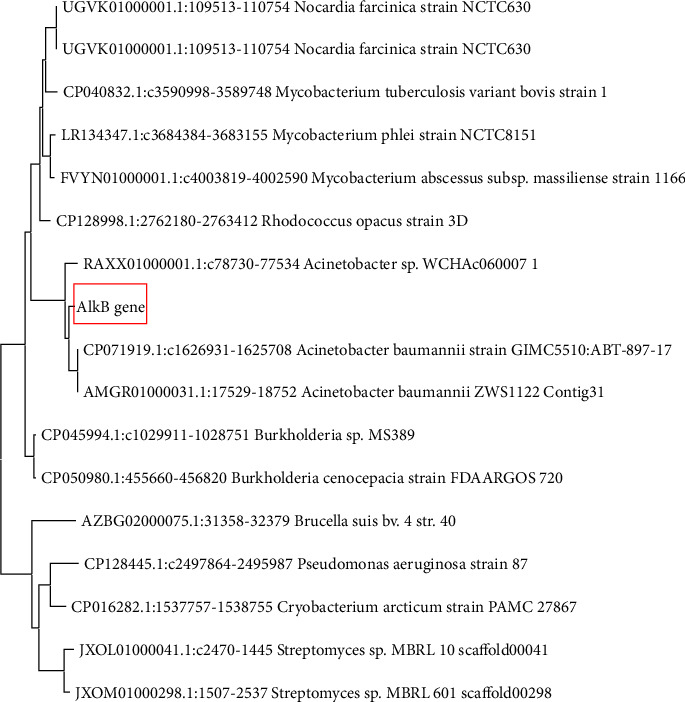
A phylogenetic tree that shows the evolutionary history inferred using the Maximum Likelihood method and Tamura–Nei model.

For phylogenetic analysis, a phylogenetic tree was constructed using the maximum likelihood method and Tamura–Nei model.  Figure 6 shows the constructed phylogenetic tree.

The novel sequence was translated and BLASTp analysis was conducted on the NCBI database. High similarity was observed with alkB genes from other bacterial species. A novel nucleotide sequence was published on the NCBI database under accession number OP959069.

## 8. Discussion

Many studies have shown that the tendency of insects like *T. molitor* to consume plastic may be linked to the digestive activity of intestinal microorganisms, which aids in the digestion process [10]. To see if the mealworms' proclivity to eat plastic was related to gut microorganisms, the gut cell suspension was cultured on a polystyrene emulsion, which provided the only carbon source for the microorganisms present. Because the polystyrene emulsion provided the nutrients, only microorganisms capable of feeding on polystyrene and thus responsible for polystyrene degradation were able to grow on the culture plates. The microorganisms isolated from the guts of *Tenebrio molitor* were confirmed to be *Klebsiella oxytoca* strains which have previously been isolated using the polymerase chain reaction (PCR) method [6]. Primers that amplify the 16S ribosomal RNA region in each of the isolates were designed to confirm that the bacteria were of *Klebsiella oxytoca* strains *Klebsiella oxytoca* ATCC 13182, *Klebsiella oxytoca* JCM 1665, and *Klebsiella oxytoca* NBRC 102593 using the provided sequences [6].

To determine the presence of the gene (alkB) responsible for plastic degradation, degenerate primers that amplify the alkB gene in bacterial isolates from mealworm guts were designed manually by selecting eleven alkB gene sequences from eleven different bacterial strains from GenBank. TCOFFE was used to align the sequences, while Jalview, an offline tool, and ConSurf, an online tool, were used to view the alignment. After aligning eleven alkB gene sequences with TCOFFE, the conserved regions were discovered. Although 99% of the bases in the conserved regions aligned well, some sequences within the conserved regions contained single-nucleotide polymorphisms. These SNPs resulted in degenerate bases in the primer sequences. Because of the degenerate bases, the manufactured primers had a melting temperature range rather than a specific temperature as with specific primers and this resulted in the application of touchdown PCR for the amplification of the gene.

To increase the chances of amplifying the alkB gene in the three isolates, two more primer sets were selected from the literature to add to the one which was designed to make them 3 primer sets [9]. These two selected primer sets were successful in amplifying the alkB gene in 49% of bacteria. The selected primers were AlkB-f and AlkB-r and Alk-BFB and Alk-BRB.

Different primers were selected because it has been reported that several bacteria have multiple alkane hydroxylases that have been shown to potentially broaden the host strain's n-alkane range [11, 12]. For example, the coexistence of AlkB and CYP153 was discovered in *Dietzia* sp. DQ12-45-1b, as well as the coexistence of multiple alkane hydroxylases in *Amycolicicoccus subflavus* DQS3-9A1T13 [13]. Some *Rhodococcus* strains were found to have multiple alkB homologous genes with varying substrate ranges and induction styles. Distribution of the alkB and CYP153 genes in the genomes of all the bacteria that had been deposited on GenBank has been reported. Multiple copies of alkB and CYP153 homologous genes were found in 20% and 36% of the alkB-containing genomes and CYP153-containing genomes, respectively. In *Rhodococcus erythropolis* SK121 and *Parvibaculum lavamentivorans* DS-1, up to six alkB homologous genes and five CYP153 homologous genes were discovered. The sequence similarities between these multiple copies of genes within one genome ranged from 27.7% to 99.7% for alkB genes and 42.4% to 100% for CYP153 genes [14].

The results from the gel in Figure 6 show that sample 3 had the alkB gene since a band was observed at 550 bp which was the expected product size with specific primers used. The other samples 1 and 2 did not show a band of amplification. This however does not necessarily mean that these bacteria do not have the alkB gene as it has been reported that a single bacterial strain can contain up to six copies of a single gene with sequence similarity as low as 28% [14]. Thus, there is a 50% chance that the gene is present, but maybe the available copy could not be amplified with this primer set used.

A phylogenetic tree was constructed using the maximum likelihood method and Tamura–Nei model in MEGA X. The results show that the alkB gene from *Klebsiella oxytoca* ATCC 13182 strain and all the bacteria in the tree share one common ancestor, but this is more closely related to the gene in *Acinetobacter*. The gene was expected to align with the alkB gene in *Klebsiella* strains because of the involvement of *Klebsiella oxytoca* in this study, but it only aligned closely with the alkB gene in *Acinetobacter* and other bacteria. This implies that no work has been done on this gene in *Klebsiella* species, resulting in the gene's deposition in databases such as GenBank. The query sequence only aligned with one alkane-1-monooxygenase gene, and the rest of the alignments were between the gene and a region in the bacteria's whole genome. This could also be explained by the lack of research on this gene in bacteria.

In addition, the use of degenerate primers can result in negative results. Degenerate primers can make optimizing PCR assays difficult because only a limited number of primer molecules in a degenerate primer mixture are complementary to the template; the melting temperature (Tm) of primer sequences can vary significantly; and the sequences of some primers can be complementary to those of others. All these issues can cause the PCR to fail, leading to the conclusion that the gene is not present when it is. A set of degenerate primers was used to amplify the alkB gene in three *Gordonia* species, and negative results were obtained until specific primers were designed and used in the PCR reaction, after which the results were positive [15]. The other *Klebsiella* oxytoca strains in this study lacked the alkB gene, as evidenced by a negative PCR. However, one can only be certain of this conclusion after using specific primers [16].

## 9. Conclusions

The work in this study outlines the discovery of the alkb gene from a *Klebsiella oxytoca* strain that was isolated from the gut of the mealworms of *T. molitor* which can feed and survive sorely on polystyrene. The polystyrene-degrading ability of the mealworms of *T. molitor* is possibly linked to bacteria of *Klebsiella oxytoca* strains that are found in the guts of the mealworms. Since the alkB gene has been linked to the degradation of polystyrene by *Klebsiella oxytoca* strains, the results provided in this study paves a way for further studies to investigate the mutual relationship between *Klebsiella oxytoca* strains and *T. molitor* in the degradation of plastic and using it as a source of carbon. Alkane-1-monooxygenase enzyme may be one of the main enzymes responsible for the degradation of polystyrene by *Klebsiella oxytoca* ATCC 13182 strain in *T. molitor*.

### 9.1. Recommendations

The authors recommend that activity tests for alkB enzyme be carried out to categorically say that the alkB enzyme is a key enzyme in the degradation of polystyrene.

## Figures and Tables

**Table 1:** DNA sequences that were used to design 16S ribosomal RNA gene-specific primers for the 3 bacterial strain.

Strain name	Sequence
*Klebsiella oxytoca* ATCC 13182	TCGTGGGGGGGCCGWGGCCCTTAMACATGCAAGCTCKAAACGGTARA-AAAAGGGGGGGAAGSTCTCGGGTGACGAGTGTTARRTAAAGGARAAGW-AAGTCTGGGAAACTGCCCGATGGARGGGGATAACTACTGGAAATTTTT-ARWTAAKACCGCATACGTCGCAAGACCAAAGAKGGGGACCTTMRGGCC-TCTTGCCATCGGATGTGCCCRATGGGATTAGCTTGTAGTGAGGTA-ACGGCTCACCTAGCGACGATCCCTAGCTGGTCTGAGAGGATGAC-CARCCACACTGGAACTGAGAC-ACGGTCCARACTCCTAGKGGAGGGCAGCRKTGGGGAATATTGCACAA-TGGGGCGRAAGCCTTGATGCAKCCATTGCC-GCCGTGTATGAAGAAGGCCTTCRGGGTTTGWAAARTT-WCTTTTCASCCGGKGAGGAAAAGGGAATTGMAGGTTTAATAAC-CCTTTTTTCCTTTRACGTTTTACCCCACCARAAAAAAGSC-WCCCKGGTTAAAYTTTYCC
*Klebsiella oxytoca* JCM 1665	ACKGGGCGGCARGCCGTACACATGCAAGTCGAACGGTAGCATAR-ARAGMTTGCTCTCGGGTGACGAGTGGCGGACGGGTGAGTAATGTCT-GGGAAACTGCCCGATGGAGGGGGATAACTAC-TGGAAACGGTAGCTAATACCGCATAA-CGTCGCAAGACCAAAGAGGGGGACCTTMGGGCCTCTTGCCATCGGATG-TGCCCAGATGGGATTAGCTTGTAGGTGAGGTAACGGCTC-ACCTAGGCGACGATCCCTAGCTGGTCTGAGAGG-ATGACCAGCCACACTGGAACTGAGACACGGTCCAGACTCCTACGGGA-GGCAGCAGTGGGGAATATTGCACAATGGGCGCAAGCCTGATGCAGCCA-TGCCGCGTGTATGAAGAAGGCCTTCGGGTTGTAAAGTACTTTCAG-CGGGGAGGAAGGG AGTGAGGTTAATAACCTTATTCATTGACGTTACCC-GCAGAAGAAGCACCGGCTAACTCCGTGCCAGCAGCCGCGGTATTACGG-AGGGTGCAGCGTTATCGGAATTACTGGGGCGTAAAG
*Klebsiella oxytoca* NBRC 102593	CAAYGGGTKKGGGGGGGYKGGCCGTACACATGSCWWGGTCGMACGGTAGTAGTRAAGAAGCTTGCTCTCGGGTGACGAGTGGCGGACGGGTGAGTAATGTCTGGGAAACTGCCCGATGGAGGGGGATAACTACTGGAAACGGTAGCTAATACCGCATAACGTCGCAAGACCAAAGAGGGGGACCTTMGGGCCTCTTGCCATCGGATGTGCCCAGATGGGATTAGCTTGTAGGTGAGGTAACGGCTCACCTAGGCGACGATCCCTAGCTGGTCTGAGAGGATGACCAGCCACACTGGAACTGAGACACGGTCCAGACTCCTACGGGAGGCAGCAGTGGGGAATATTGCACAATGGGCGCAAGCCTGATGCAGCCATGCCGCGTGTATGAAGAAGGCCTTCGGGTTGTAAAGTACTTTCAGCGGGGAGGAAGGGAGTGAGGTTAATAACCTTATTCATTGACGTTACCCGCAGAAGAAGCACCGGCTAACTCCGTGCCAGCAGCCGCGGTAATACGGAGGGGGTGCAAAGCCGTTTAAATCC GGGGAAT

**Table 2:** The species name and strain and the accession numbers of the alkB gene sequences used for the alignment.

Species name	Accession number
*Gordonia alkanivorans strain DSM 44369*	GU130258.1
*Nocardia* sp. *SoB clone B78*	EF437965.1
*Rhodococcus* sp. *strain CS*	MT024647.1
*Bacterium alkW43*	DQ287998.1
*Rhodococcus* sp. *strain N1*	MT024641.1
*Rhodococcus fascians*	AJ301873.1
*Acinetobacter calcoaceticus strain M10*	KF584908.1
*Bradyrhizobiaceae bacterium MS228e*	JN616314.1
*Nocardia* sp. *SoB clone B78*	EF437965.1
*Gordonia rhizosphera strain DSM 44383*	GU130265.1
*Uncultured soil bacterium clone GO0VNXF07H1URE*	JF406820.1

**Table 3:** Forward and the reverse primer sequences designed to amplify the 16S ribosomal RNA of the *Klebsiella oxytoca* NBRC 102593 strain, *Klebsiella oxytoca* JCM 1665 strain, and *Klebsiella oxytoca* ATCC 13182 strain and alkB gene in *Klebsiella oxytoca* strains.

Target	Primer sequence (5′-3′)	*T*_*m*_ (°C)	GC (%)	Amplicon size (bp)
*Klebsiella oxytoca* NBRC 102593	NBRC forward: GATGACCAGCCACACTGGAA	59.96	55	247
	NBRC reverse: GCTGCACCCTCCGTAATACC	60.53	60	

*Klebsiella oxytoca* ATCC 13182	ATCC forward: TCTCGGGTGACGAGTGTTA	57.67	52.63	329
	ATCC reverse: CTTCATACACGGCGGCAATG	59.97	55	

*Klebsiella oxytoca* JCM 1665	JCM forward: CCAGCCACACTGGAACTGAG	60.6	60.0	262
	JCM reverse: TCCCCGGATTTAAACGGCTTTG	61.46	50.0	

alkB gene	Deg-f forward: CACTTCTACRTCGATCAC	42.81	50	338
	Deg-r reverse: CCRTAGTGCTCGAGGTAG	47.69	58.82	

**Table 4:** Similarity of the alkB sequence in *Klebsiella oxytoca* ATCC with the alkB gene sequences in 10 selected bacteria.

Accession number	E value	Per. Ident	Region of alignment
CP028800.2	1.00*E* − 135	96.07	https://www.ncbi.nlm.nih.gov/protein/1379042730
CP019041.1	1.00*E* − 135	96.07	https://www.ncbi.nlm.nih.gov/protein/2215689426
CP094445.1	1.00*E* − 135	96.07	https://www.ncbi.nlm.nih.gov/protein/1130059488
CP078018.1	1.00*E* − 135	96.07	https://www.ncbi.nlm.nih.gov/protein/2260151666
CP069426.1	1.00*E* − 135	96.07	https://www.ncbi.nlm.nih.gov/protein/2070261387
CP068253.1	1.00*E* − 135	96.07	https://www.ncbi.nlm.nih.gov/protein/1958834060
CP024632.1	5.00*E* − 134	95.74	https://www.ncbi.nlm.nih.gov/protein/1275534167
KY417159.1	5.00*E* − 134	95.74	https://www.ncbi.nlm.nih.gov/protein/1196819846
CP099548.1	5.00*E* − 134	95.74	https://www.ncbi.nlm.nih.gov/protein/2260151666
CP078042.1	5.00*E* − 134	95.74	https://www.ncbi.nlm.nih.gov/protein/2070244498

## Data Availability

The datasets generated during the current study are available in the (NCBI) repository (Banklt2650355 BSeq#1 OP959069).

## References

[1] Brandon A. M., Gao S. H., Tian R. (2018). Biodegradation of polyethylene and plastic mixtures in mealworms (larvae of *T. molitor*) and effects on the gut microbiome. *Environmental Science and Technology*.

[2] Mohanan N., Montazer Z., Sharma P. K., Levin D. B. (2020). Microbial and enzymatic degradation of synthetic plastics. *Frontiers in Microbiology*.

[3] Kesti S. S., Ct S. (2019). The role of insects and microorganisms in plastic biodegradation: a comprehensive review. *International Journal of Scientific Research in Biological Sciences*.

[4] Hou L., Majumder E. L. W. (2021). Potential for and distribution of enzymatic biodegradation of polystyrene by environmental microorganisms. *Materials*.

[5] Urbanek A. K., Rybak J., Wróbel M., Leluk K., Mironczuk A. M. (2020). A comprehensive assessment of microbiome diversity in *T. molitor* fed with polystyrene waste. Env. *Environmental Pollution*.

[6] Machona O., Chidzwondo F., Mangoyi R. (2022). *Tenebrio molitor*: possible source of polystyrene-degrading bacteria. *BMC Biotechnology*.

[7] Gil-Jasso N. D., Giles-Mazón E. A., Soriano-Giles G., Reinheimer E. W., Varela-Guerrero V., Ballesteros-Rivas M. F. (2022). A methodology for recycling waste expanded polystyrene using flower essential oils. *Fuel*.

[8] Smits T. H. M., Balada S. B., Witholt B., van Beilen J. B. (2002). Functional analysis of alkane hydroxylases from Gram-negative and Gram-positive bacteria. *Journal of Bacteriology*.

[9] Jurelevicius D., Alvarez V. M., Peixoto R., Rosado A. S., Seldin L. (2013). The use of a combination of alkB primers to better characterize the distribution of alkane-degrading bacteria. *PLoS One*.

[10] Yang S. S., Wu W. M. (2020). Biodegradation of plastics in Tenebrio genus (mealworms). Handbook of env. *Chem*.

[11] Amouric A., Quéméneur M., Grossi V., Liebgott P. P., Auria R., Casalot L. (2010). Identification of different alkane hydroxylase systems in Rhodococcus ruber strain SP2B, an hexane-degrading actinomycete. *Journal of Applied Microbiology*.

[12] Li Y. P., Pan J. C., Ma Y. L. (2020). Elucidation of multiple alkane hydroxylase systems in biodegradation of crude oil *n*‐alkane pollution by *Pseudomonas aeruginosa* DN1. *Journal of Applied Microbiology*.

[13] Wang X. B., Chi C. Q., Nie Y. (2011). Degradation of petroleum hydrocarbons (C6-C40) and crude oil by a novel *Dietzia* strain. *Bioresource Technology*.

[14] Nie Y., Wu X. L., Chi C. Q. (2014). Diverse alkane hydroxylase genes in microorganisms and environments. *Scientific Reports*.

[15] Shen F. T., Young L. S., Hsieh M. F., Lin S. Y., Young C. C. (2010). Molecular detection and phylogenetic analysis of the alkane 1-monooxygenase gene from Gordonia spp. *Systematic & Applied Microbiology*.

[16] Yang X., Scheffler B. E., Weston L. A. (2006). Recent developments in primer design for DNA polymorphism and mRNA profiling in higher plants. *Plant Methods*.

[17] Vrancken B., Lemey P., Rambaut A. (2015). Simultaneously estimating evolutionary history and repeated traits phylogenetic signal: applications to viral and host phenotypic evolution. *Methods in Ecology and Evolution*.

